# Metabolomics Studies on Cytoplasmic Male Sterility during Flower Bud Development in Soybean

**DOI:** 10.3390/ijms20122869

**Published:** 2019-06-12

**Authors:** Xianlong Ding, Xuan Wang, Qiang Li, Lifeng Yu, Qijian Song, Junyi Gai, Shouping Yang

**Affiliations:** 1 Soybean Research Institute, National Center for Soybean Improvement, Key Laboratory of Biology and Genetic Improvement of Soybean (General, Ministry of Agriculture), State Key Laboratory of Crop Genetics and Germplasm Enhancement, Jiangsu Collaborative Innovation Center for Modern Crop Production, College of Agriculture, Nanjing Agricultural University, Nanjing 210095, China; xlding2012@njau.edu.cn (X.D.); 2014101141@njau.edu.cn (X.W.); 2017101147@njau.edu.cn (Q.L.); 2017101148@njau.edu.cn (L.Y.); sri@njau.edu.cn (J.G.); 2 Soybean Genomics and Improvement Laboratory, Beltsville Agricultural Research Center, USDA-ARS, Beltsville, MD 20705, USA; Qijian.Song@ARS.USDA.GOV

**Keywords:** soybean CMS-N8855, flower bud development, metabolomics, reactive oxygen species, non-enzymatic ROS scavenging system, enzymatic ROS scavenging system

## Abstract

Abnormal reactive oxygen species (ROS) may mediate cytoplasmic male sterility (CMS). To observe the effect of ROS on soybean CMS, metabolite content and antioxidant enzyme activity in the flower buds between soybean N8855-derived CMS line and its maintainer were compared. Of the 612 metabolites identified, a total of 74 metabolites were significantly differentiated in flower buds between CMS line and its maintainer. The differential metabolites involved 32 differential flavonoids, 13 differential phenolamides, and 1 differential oxidized glutathione (GSSG) belonging to a non-enzymatic ROS scavenging system. We observed lower levels of flavonoids and antioxidant enzyme activities in flower buds of the CMS line than in its maintainer. Our results suggest that deficiencies of enzymatic and non-enzymatic ROS scavenging systems in soybean CMS line cannot eliminate ROS in anthers effectively, excessive accumulation of ROS triggered programmed cell death and ultimately resulted in pollen abortion of soybean CMS line.

## 1. Introduction

Cytoplasmic male sterility (CMS) is a maternally inherited trait that has been found in over 150 plant species [1]. CMS is associated with abnormal recombination of mitochondrial genome [2,3,4]. Mitochondria are vital for producing ATP via the mitochondrial electron transport chain of the respiratory complexes [4], and its genome reorganization often leads to energy deficiency and abnormal accumulation of reactive oxygen species (ROS) in plant anther because mitochondria is the main organelle generating ROS [5,6]. ROS play an important role in cell signaling of plant pollen, its overproduction can cause damage to lipids, proteins and DNA, inhibit enzyme activity, activate programmed cell death (PCD) pathway (premature or delayed), and ultimately affect plant growth [7,8,9,10,11,12,13].

Many reports have suggested that abnormal ROS levels and deficiency of ROS scavenging system are associated with male sterility in crops including rice [14,15], cotton [6], pepper [16], and maize [17]. There are two ROS scavenging systems in plants [13,16,18]. Enzymatic ROS scavenging system usually consists of enzymes such as super oxide dimutase (SOD), catalase (CAT), peroxidase (POD) [14,18,19], and they are all abnormally expressed during pollen development in plant with CMS [6,14,15,16,17]. The non-enzymatic ROS scavenging system consists mainly of some secondary metabolites, such as major cellular redox buffers ascorbate (AsA) and glutathione (GSH), anthocyanins, flavonoids, and phenolamides [13,20,21]. These metabolites play a crucial role in pollen development and protection from ROS stresses [13,18,20,22,23,24].

N8855 is one of the important donors of male-sterile cytoplasm in soybean [25]. Our previous study revealed that male sterility in soybean CMS line NJCMS1A (with N8855 cytoplasm) is related to ROS response disorders [26]. However, the molecular mechanisms underlying ROS induced damage in flower buds of soybean CMS remain to be fully elucidated. Metabolomics is a powerful tool for understanding the mechanisms of metabolic regulation during flower, anther, and pollen development [18,23,27,28], it also provides new insights into the metabolism related to ROS stress during plant growth and development, such as pollen development during heat stress response [23]. In this study, we compared the flower bud metabolite content and antioxidant enzyme activities between soybean N8855-derived CMS line and its maintainer. Our results showed that three down-regulated metabolites (flavonoids, phenolamides, and oxidized glutathione (GSSG)) and two low-activity antioxidant enzymes (CAT and POD) might be associated with ROS scavenging disorder and pollen abortion in soybean CMS.

## 2. Results

### 2.1. Metabolite Profiling of Soybean Flower Buds

**Figure 1:**
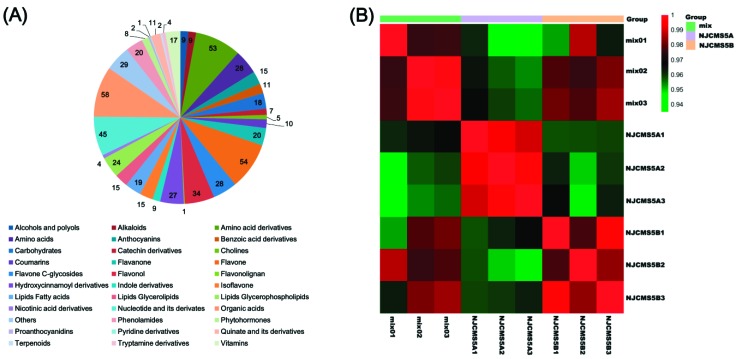
The distribution of identified metabolites in flower buds of NJCMS5A and NJCMS5B and the repeated correlation assessment (RCA) analysis. (**A**) The distribution of identified 612 metabolites in flower buds of NJCMS5A and NJCMS5B; (**B**) The RCA analysis of NJCMS5A, NJCMS5B, and mix. Mix, quality control sample.

Metabolite analysis identified 612 metabolites in N8855-derived CMS line NJCMS5A and its maintainer NJCMS5B (Table S1). The 612 metabolites could be assigned into 33 categories, including 9 alcohols and polyols, 9 alkaloids, 53 amino acid derivatives, 28 amino acids, 15 anthocyanins, 11 benzoic acid derivatives, 18 carbohydrates, 7 catechin derivatives, 5 cholines, 10 coumarins, 20 flavanone, 54 flavone, 28 flavone C-glycosides, 34 flavonol, 1 flavonolignan, 27 hydroxycinnamoyl derivatives, 9 indole derivatives, 15 isoflavone, 19 lipids fatty acids, 15 lipids glycerolipids, 24 lipids glycerophospholipids, 4 nicotinic acid derivatives, 45 nucleotide and its derivates, 58 organic acids, 20 phenolamides, 8 phytohormones, 2 proanthocyanidins, 1 pyridine derivatives, 11 quinate and its derivatives, 2 terpenoids, 4 tryptamine derivatives, 17 vitamins, and 29 other metabolites (Figure 1A, Table S1). As showed in Figure 1B, the *R*^2^ ˃ 0.93 and close to 1, indicating that there was a good repeatability of each line.

### 2.2. Principal Component Analysis (PCA) and Orthogonal Projections to Latent Structures-Discriminant Analysis (OPLS-DA) for the NJCMS5A Group Versus NJCMS5B Group

**Figure 2:**
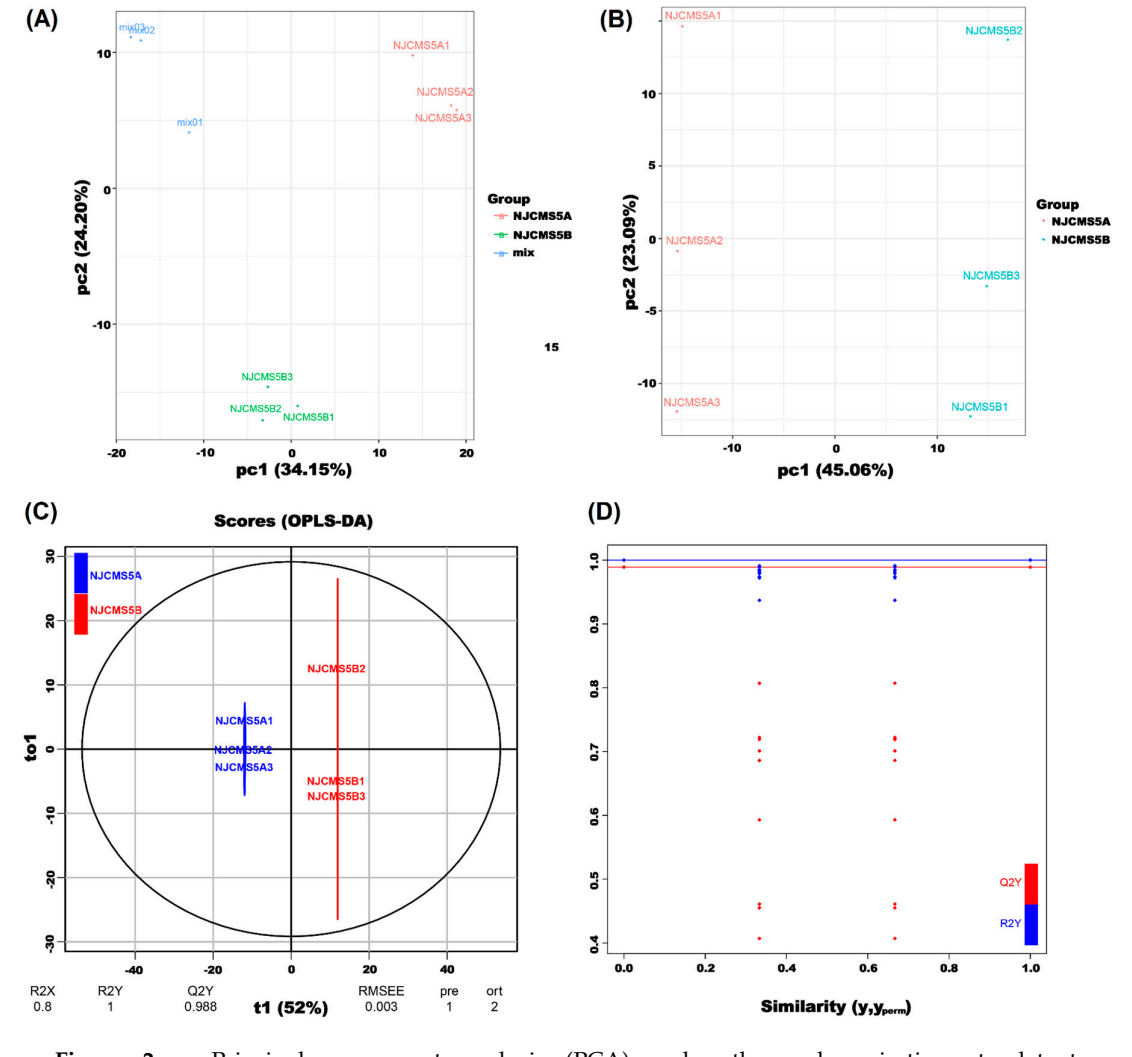
Principal component analysis (PCA) and orthogonal projections to latent structures-discriminant analysis (OPLS-DA). (**A**) PCA score scatter plot for mass spectrum data of NJCMS5A, NJCMS5B, and mix (quality control sample). The *x* axis and *y* axis indicate the PC1 and PC2, respectively; (**B**) Score scatter plot of the PCA model for the NJCMS5A versus NJCMS5B. The *x* axis and *y* axis indicate the PC1 and PC2, respectively; (**C**) Score scatter plot of the OPLS-DA model for the NJCMS5A versus NJCMS5B. R2X and R2Y represent the explanatory rate of the model to *x* and *y* matrices, respectively, and Q2 represents the predictive ability of the model. The three indicators are close to 1, which indicates the model is more stable and reliable; (**D**) Permutation test of the OPLS-DA model for the NJCMS5A versus NJCMS5B. The horizontal lines correspond to R2 and Q2 values of the original model. The red and blue points indicate the R2’ and Q2’ values obtained by the permutation test.

The PCA score scatter plots for all samples (including mix samples) are shown in Figure 2A, where the distinction between the NJCMS5A group, the NJCMS5B group, and the mix group was significant. In order to find correlating metabolic changes, PCA was performed with all 612 metabolites. The first two PCs accounted for 45.06% and 23.09% of the variance (Figure 2B), respectively, which indicated that significant differences between NJCMS5A and NJCMS5B existed. The OPLS-DA model was applied to visualize the metabolic alterations between NJCMS5A and NJCMS5B, where the R2X, R2Y, and Q2 values were 0.8, 1 and 0.988, respectively (Figure 2C). This result showed that the OPLS-DA model was stable and efficient. In the permutation test, both R2’ and Q2’ were smaller than R2 and Q2 of the OPLS-DA model, which showed that the model is meaningful (Figure 2D). Overall, the OPLS-DA model provided a credible explanation of the difference between NJCMS5A group and NJCMS5B group. Most importantly, it provides information that we can screen the differential metabolites based on variable importance in projection (VIP) analysis.

### 2.3. Metabolic Changes in Flower Bud of Soybean CMS Line

**Figure 3:**
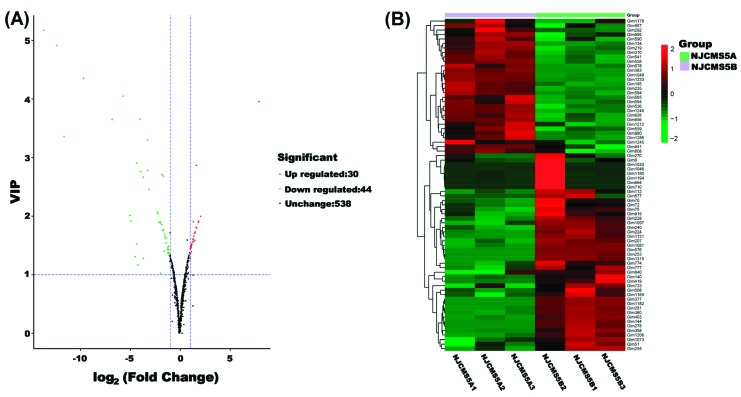
Volcano plot and heat map analysis of differential metabolites between NJCMS5A and NJCMS5B. (**A**) Volcano plot of differential metabolites between NJCMS5A and NJCMS5B. Red point, green point, and black point indicate the metabolites that were significantly up-regulated, significantly down-regulated, and non-significantly different, respectively; (**B**) Heat map analysis of 74 differential metabolites between NJCMS5A and NJCMS5B. The values of differential metabolites were normalized and shown as a color scale. The high and low metabolite levels were represented as reddish and greenish scales, respectively.

**Figure 4:**
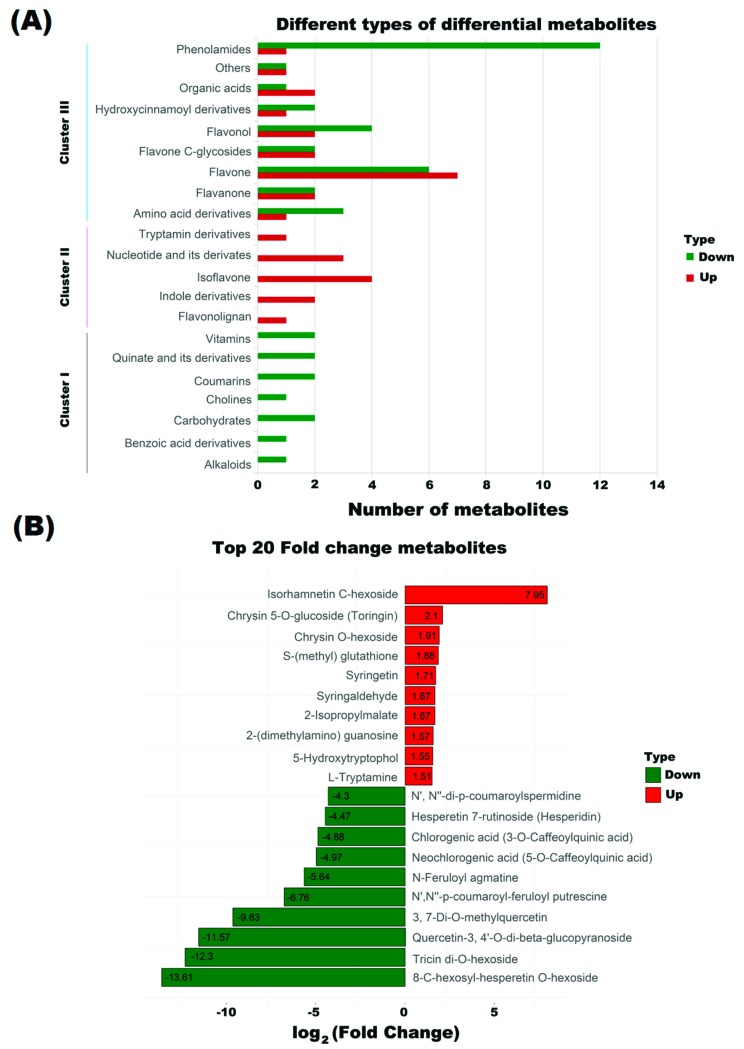
Cluster of different types of differential metabolites for NJCMS5A and NJCMS5B. (**A**) Cluster of different types of differential metabolites between NJCMS5A and NJCMS5B; (**B**) Top 10 up-regulated metabolites and top 10 down-regulated metabolites.

Based on OPLS-DA results, 74 metabolites were differential between NJCMS5A and NJCMS5B (|Fold change| ≥2 and VIP ≥ 1), including 30 up-regulated and 44 down-regulated metabolites in flower buds of NJCMS5A compared with that of NJCMS5B (Table S2, Figure 3). The metabolites were classified into three clusters (Figure 4A). Cluster I included 1 alkaloids, 1 benzoic acid derivatives, 2 carbohydrates, 1 cholines, 2 coumarins, 2 quinate and its derivatives, and 2 vitamins. The metabolites in this cluster were all down-regulated in soybean CMS line. However, the metabolites in cluster II were all up-regulated, including 1 flavonolignan, 2 indole derivatives, 4 isoflavone, 3 nucleotide and its derivates, and 1 tryptamine derivatives. Metabolites in cluster III were both up-regulated and down-regulated. The cluster contained 9 categories, including 4 amino acid derivatives, 4 flavanone, 13 flavone, 4 flavone C-glycosides, 6 flavonol, 3 hydroxycinnamoyl derivatives, 3 organic acids, 2 others, and 13 phenolamides.

**Figure 5:**
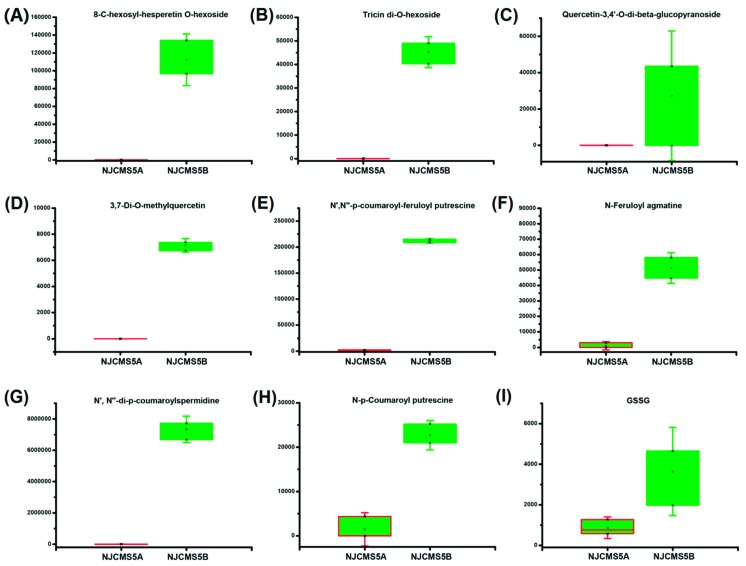
Box plots of differential metabolites involved in non-enzymatic ROS scavenging system of NJCMS5A and NJCMS5B. (**A**) 8-C-hexosyl-hesperetin *O*-hexoside; (**B**) Tricin di-*O*-hexoside; (**C**) Quercetin-3,4′-*O*-di-β-glucopyranoside; (**D**) 3,7-Di-*O*-methylquercetin; (**E**) N′,N″-p-coumaroyl-feruloyl putrescine; (**F**) *N*-Feruloyl agmatine; (**G**) *N*′,*N*″-di-p-coumaroylspermidine; (**H**) *N*-p-Coumaroyl putrescine; (**I**) GSSG-Glutathione oxidized.

**Figure 6:**
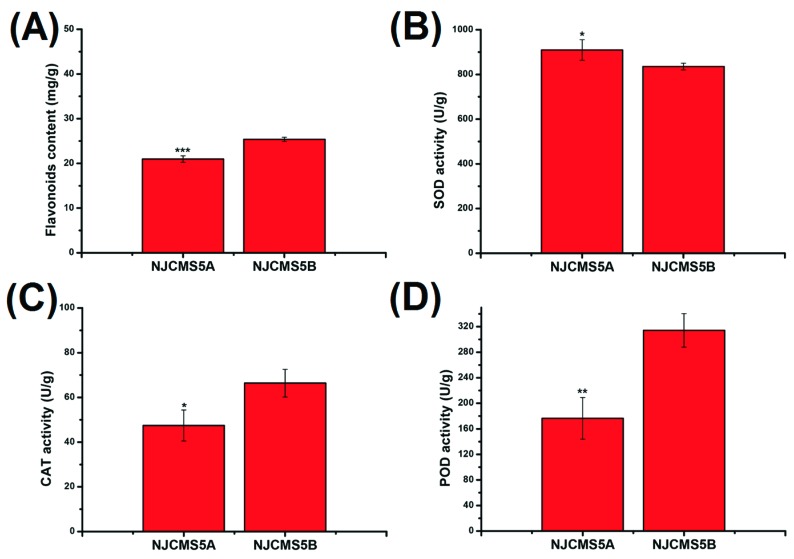
Changes of flavonoid content and antioxidant enzyme activities in flower buds of NJCMS5A and NJCMS5B. (**A**) Flavonoids; (**B**) SOD–Super oxide dimutase; (**C**) CAT–Catalase; (**D**) POD–Peroxidase. Asterisk indicates statistical differences, * *p* < 0.05; ** *p* < 0.01; *** *p* < 0.001.

Of the 32 differential flavonoids with fold difference ranging from −12,477.78 to 247.04, a total of 18 were up-regulated and 14 down-regulated in NJCMS5A vs. NJCMS5B (Table 1). Figure 4B lists the top four down-regulated flavonoids, included 8-C-hexosyl-hesperetin O-hexoside (−12,477.78 fold), tricin di-*O*-hexoside (−5025.93 fold), quercetin-3,4’-*O*-di-β-glucopyranoside (−3033.67 fold) and 3,7-Di-*O*-methylquercetin (−794.44 fold). These four differential metabolites were related to hexoside and quercetin, and were only detected in NJCMS5B (Table S2; Figure 5A–D). Flavonoids content was significantly lower in NJCMS5A than NJCMS5B (Figure 6A).

Phenolamides constituted a major group of identified metabolites in this study, and most of them (13/20) were significantly different between NJCMS5A and NJCMS5B (Table S2). The 12 down-regulated phenolamides in NJCMS5A were divided into three groups: spermidine, putrescine, and agmatine (Table 2). The top four down-regulated phenolamids were *N*′,*N*″-p-coumaroyl-feruloyl putrescine (−108.53 fold), N-Feruloyl agmatine (−50.00 fold), *N*′, *N*″-di-p-coumaroylspermidine (−19.65 fold), and *N*-p-Coumaroyl putrescine (−15.56 fold) (Figure 5E–H). Differential metabolites with the same characteristics, such as *N*′,N″-di-p-coumaroylspermidine, *N*-Feruloyl agmatine, *N*-Caffeoylspermidine, and *N*′,*N*″-Feruloyl,caffeoylspermidine were grouped into phenolamides and were all down-regulated in NJCMS5A (Figure 3B and Figure 4A). These results indicate that some groups might play a unique role in the formation of soybean CMS.

Table S2 lists the significant differences of amino acid derivatives, 3 of them were down-regulated in NJCMS5A vs. NJCMS5B, and GSSG was the most down-regulated differential metabolites (Figure 5I).

### 2.4. Altered Functional Pathways in Soybean CMS Line

**Figure 7:**
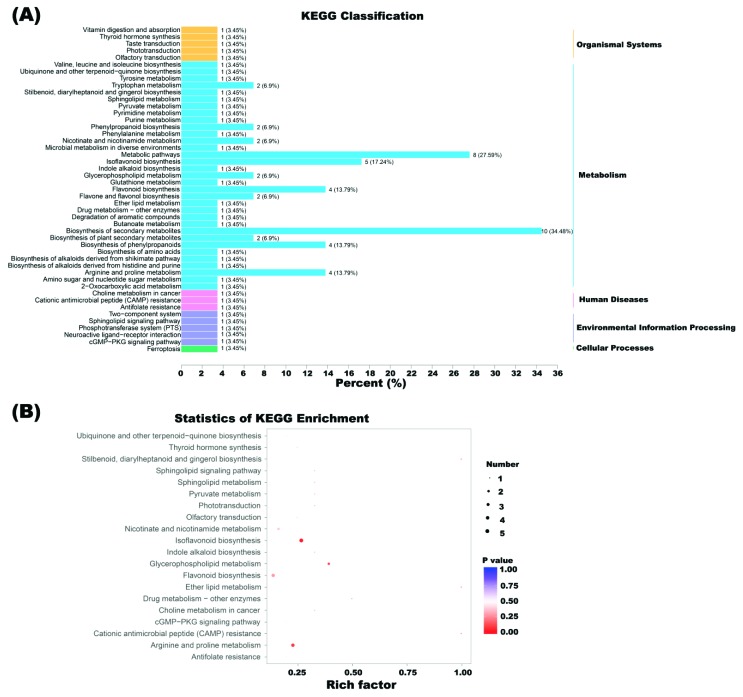
Cluster of different types and pathway analysis of differential metabolites for NJCMS5A and NJCMS5B. (**A**) KEGG classification of 47 pathways from 29 differential metabolites. The *x* axis indicates the proportion and number of metabolites annotated to the pathway, and the *y* axis indicates name of the KEGG metabolic pathway; (**B**) Statistics of KEGG enrichment. The *x* axis indicates the rich factor corresponding to each pathway, and the *y* axis indicates name of the KEGG metabolic pathway. The color of the point represents the *p*-values of the enrichment analysis. The size and color of bubbles represent the number and degree of enrichment of different metabolites, respectively.

To further illustrate the interaction of differential metabolites involved in flower bud development of soybean CMS line, pathways of 74 differential metabolites were analyzed at KEGG website. A total of 29 metabolites were mapped to 47 KEGG pathways (Table S3), including biosynthesis of secondary metabolites, metabolic pathways, isoflavonoid biosynthesis, biosynthesis of phenylpropanoids, flavonoid biosynthesis, arginine and proline metabolism, tryptophan metabolism, glycerophospholipid metabolism, nicotinate and nicotinamide metabolism, flavone and flavonol biosynthesis, and biosynthesis of plant secondary metabolites etc (Figure 7A). The top five metabolic pathways with the highest *p*-values in metabolic pathway enrichment analysis were isoflavonoid biosynthesis, glycerophospholipid metabolism, arginine and proline metabolism, stilbenoid, diarylheptanoid and gingerol biosynthesis, and ether lipid metabolism (Figure 7B, Table S4).

In addition, other flavonoids metabolic pathways such as flavonoid biosynthesis, flavone, and flavonol biosynthesis were identified (Table S4). Enrichment analysis is consistent with the fact that there was 32 differential flavonids (43.24% of the total number of differential metabolites) and 14 flavonids of them were down-regulated in NJCMS5A compared with NJCMS5B (Table S2).

### 2.5. Activities of ROS Scavenging Enzymes

Of the three major antioxidant enzymes, SOD, POD, and CAT, activities for the POD and CAT were significantly decreased (Figure 6C,D), while SOD activity was significantly increased in NJCMS5A than NJCMS5B (Figure 6B).

## 3. Discussion

### 3.1. Differential Metabolites between Soybean CMS Line and Its Maintainer

Although metabolites during flower, anther, and pollen development in rice [18], tomato [23], wheat [27], and pear [28] have been reported, changes in metabolites associated with pollen abortion in CMS plant (except cotton and potato CMS) is limited [29,30].

In this study, 74 differential metabolites were identified in the comparison of soybean N8855-derived CMS line and its maintainer (Figure 3, Table S2). Like in cotton CMS line, most metabolites were down-regulated than those in maintainer [30], which may cause pollen abortion in soybean NJCMS5A. Previous transcriptome and proteome studies have found that many down-regulated genes and proteins in the flower buds of soybean CMS line were involved in the carbohydrate metabolism pathway [26,31]. In this study, all of the two differential carbohydrate metabolites were down-regulated (Table S2), suggesting that the down-regulation of related genes in soybean CMS line hindered the synthesis of carbohydrate metabolism-related proteins and metabolites, leading to insufficient energy supply and affecting pollen development. Flavonoids and phenolamides were the most two differential metabolites, many studies indicated that they may facilitate the scavenging of ROS and pollen development [20,23].

### 3.2. Disorder of Metabolites in Non-enZymatic ROS Scavenging System of Soybean CMS Line

Abnormal ROS status is associated with pollen abortion in many CMS plants [6,14,16]. A total of 46 differential metabolites (constituting 62.16% of the total differential metabolites) response to oxidation tolerance were found, these includes 32 differential flavonoids, 13 differential phenolamides, and 1 GSSG (Table S2) which might belong to the non-enzymatic ROS scavenging system.

Flavonoids are one of the powerful secondary metabolites that protect plant against ROS stresses [32]. In plant, flavonoids can be oxidized by POD, it scavenge H_2_O_2_ in the phenolic/AsA/POD system [13]. In this study, of the 32 differential flavonoids between flower buds from soybean CMS line and its maintainer, 43.75% were down-regulated (Table 1 and Table S2; Figure 4A) and four (tricin di-*O*-hexoside, 8-*C*-hexosyl-hesperetin *O*-hexoside, quercetin-3,4′-*O*-di-beta-glucopyranoside and 3,7-Di-*O*-methylquercetin) were even not detected in the CMS line (Table S2; Figure 5A–D). Quercetin is one of the most important flavonoid forms in plant, and previous researches in *Petunia* and tobacco showed that it is essential for pollen tube germination and growth [33,34]. Flavonoids are present in pollen of many plants, and play an important role in pollen development, especially on pollen germination or pollen tube growth [23,35]. However, its production depends on the chalcone synthase (CHS), the first enzyme in the flavonoid pathway [36]. After decades of study, researchers have found that antisense suppression of CHS would result in pollen sterility in many plants, including *Petunia* [33,37,38], maize [39], and tomato [36]. In previous study, a down-regulated of CHS involved in the flavonoid synthesis pathway was detected during soybean CMS flower bud development [26], in this study, we observed a large decrease in flavonoid content during flower bud development in the CMS line vs. its maintainer (Figure 6A). The down-regulation of CHS in soybean CMS line may reduce the synthesis of flavonoids, which consequently reduces the scavenge efficiency of ROS, and lead to ROS outbreak and pollen abortion. The decrease of flavonoids content may also result in the inability of pollen germination in soybean CMS line (Figure S1C). The relationship between CHS expression and flavonoids synthesis, and mechanism of flavonoids participating in the scavenging ROS and regulating fertility need further research.

**Figure 8:**
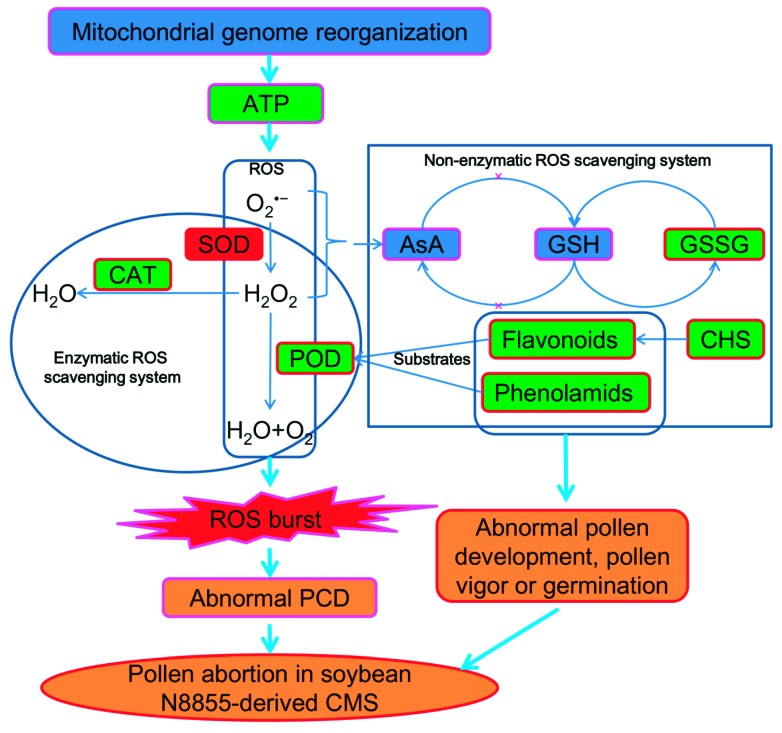
A proposed model for ROS burst and ROS scavenging system deficiency in soybean CMS-N8855. Pink and bright red boxes indicate a conclusion supported by experimental evidence in reports from previous studies and this study, respectively. The up-regulated and down-regulated of enzyme activity or metabolite content are in red and green, respectively.

Phenolamides are products of polyamine catabolism and constitute a major group of secondary metabolites that are abundant in reproductive organs of flowering plants [20]. The phenolamide forms detected in flower bud of soybean were coumaroyl, caffeoyls, and feruloyl groups (Table S2). In this study, 65% of phenolamides were differential metabolites, 12 of them (92.31%) were down-regulated in flower buds of soybean N8855-derived CMS line compared to its maintainer (Table S2). Phenolamides were proposed to be a necessary component to floral induction and development and have been detected in the anthers or pollen of *Dicots* [40,41]. Phenolamides first accumulate in the upper leaves and subsequently in floral organs [42,43], they are essential for pollen development, viability, or germination. In maize, their absence in anthers led to male sterility [44]. The di-p-coumaroylspermidine is classified as an important phenolamides in the development of flower organs, and it is considered as markers of male fertility [42,45]. In this study, *N*′,*N*″-di-p-coumaroylspermidine was classified as di-p-coumaroylspermidine and was highly reduced in flower buds of soybean CMS line (Figure 5G), suggesting that it might be closely related to pollen fertility in soybean. Phenolamides is also known to have antioxidant and radical scavenging properties, they are substrates for POD, and to eliminate H_2_O_2_ [20]. The blocked synthesis of phenolamides may restrict the scavenging of ROS and affect pollen development, pollen viability, or germination (Figure 8). It is unclear what influences the synthesis of phenolamides and how the lack of phenolamides results in pollen sterility of soybean CMS line.

AsA and GSH are important protective antioxidant substances in plant, they can effectively remove ROS through AsA–GSH cycle [13]. As an eliminator, AsA scavenges free radicals by reacting with ROS directly with primary antioxidants, while GSH can react chemically with O_2_^•−^ and H_2_O_2_ as a free radical scavenger, yielding GSSG [13,46]. GSSG is oxidized from GSH and participate in regeneration of AsA, via the AsA–GSH cycle [13,46]. GSSG was the most decreased amino acid derivatives (−4.17 fold) in flower buds of soybean N8855-derived CMS line (Table S2; Figure 5I), which might hinder the normal regeneration of GSH in flower buds of soybean CMS line. Analysis of the transcriptomics and proteomics data from soybean flower bud identified glutathione S-transferase 7 gene and gamma-glutamyltranspeptidase 1-like protein corresponding to the glutathione metabolism and both were down-regulated in N8855 derived CMS line (Table S5) [26,31]. This reflects the abnormality of AsA–GSH cycle in soybean CMS-N8855 and the result is consistent with the decrease of GSSG in our metabolic observations.

### 3.3. Altered Enzymatic ROS Scavenging System Exist in Flower Buds of Soybean CMS Line

In addition to the non-enzymatic ROS scavenging system, to maintain ROS homeostasis, there is also an efficient and rapid reaction enzymatic system in plants and animals [14,18,19]. Of the three main enzymes, SOD prevents ROS burst by reducing O_2_^•−^ into O_2_ and H_2_O_2_ [14,46,47], which are then decomposed by POD and CAT. In this study, the SOD activity in flower buds of NJCMS5A was higher than that of NJCMS5B (Figure 6B), which was consistent with the research on pepper CMS [16]. On the contrary, the POD and CAT were significantly down-regulated during flower bud development in soybean NJCMS5A (Figure 6C,D). The high activity of SOD is a natural response of soybean CMS line to the increase of ROS, as that SOD forms the first line to defense against ROS in plant [14,46,47]. However, the scavenging rate of O_2_^•−^ by higher activity of SOD is less than that of its production. With the continuous production of H_2_O_2_ and the decline of POD and CAT activity, ROS burst occurs.

### 3.4. Proposed Model for ROS Burst and ROS Scavenging System Deficiency in Soybean CMS-N8855

CMS is caused by the combination of recombinant mitochondrial gene and nuclear gene, which lead to energy deficiency or the aberrant PCD [3,4,14,48,49,50]. Mitochondria are vital for producing ATP, and the reorganization of mitochondria genome often leads to energy deficiency in plant anthers, which induced a series of changes such as abnormal accumulation of ROS in plant anther, because mitochondria are the main source of forming ROS and it provide energy for cell response to ROS [4,5,6,51,52]. ROS plays an important role in cell signaling, its overproduction affects progression of PCD (premature or delayed) [7,8,9,10], premature PCD of the tapetal cells lead to male sterility in many plants [4,53,54].

Previous studies have found significant differences in mitochondrial genes between soybean N8855-derived CMS line and its maintainer, there was recombination in soybean CMS mitochondrial genome [55]. Comparative transcriptome and proteomics analysis have found that there were many differentially expressed genes and proteins between soybean CMS line and its maintainer, soybean CMS-N8855 might be associated with energy disorder and ROS burst [26,31,56]. Our previous research also showed that tapetum cells developed abnormally and aberrant PCD might exist in soybean CMS-N8855 [31,56,57], which might be induced by ROS burst.

Based on our previous reports and this study, we proposed a model to integrate the ROS burst and ROS scavenging system deficiency in soybean CMS-N8855 (Figure 8). In this model, the mitochondrial function of soybean N8855-derived CMS line is disordered by the mitochondrial genome rearrangement, which then leads to the decrease of ATP production and the increase of ROS content. The most lethal was that the deficiencies of enzymatic and non-enzymatic ROS scavenging systems lead to inadequate removal of ROS, followed by induction of abnormal PCD and pollen abortion in soybean CMS plant (Figure 8), a cross-action between enzymatic and non-enzymatic ROS scavenging systems may occur in the process of ROS scavenging. For example, both flavonoids and phenolamides could be oxidized by POD, and scavenge H_2_O_2_ [13,20]. The reduction or lack of non-enzymatic ROS scavenging metabolites such as flavonoids and phenolamides may also affect pollen development, pollen vigor or germination (Figure 8).

## 4. Materials and Methods

### 4.1. Plant Materials

A CMS line NJCMS5A (with the cytoplasm of N8855) and its maintainer NJCMS5B [58,59,60,61] were planted in the summer of 2017 at the Dangtu Experimental Station, National Center for Soybean Improvement, Nanjing Agricultural University, Dangtu, Anhui, China. Because it is very difficult to determine pollen development stage of the flower buds in soybean, the flower buds of different sizes were collected and mixed from NJCMS5A and NJCMS5B plants, respectively, then immediately frozen in liquid nitrogen and stored at −80 °C for further use. Flower buds from each genotype were collected from at least three individual plants, and serve as three biological replicates for both NJCMS5A and NJCMS5B. Mix sample (quality control sample) was prepared by mixing extracts of NJCMS5A and NJCMS5B.

### 4.2. Plant Traits Investigation

The male-sterile and fertile plants were identified using four methods including the dehiscence of anthers (Figure S1A), I_2_-KI staining, germination rate of pollens (Figure S1B,C), and plant morphology at maturity (Figure S1D). The anther dehiscence, 1% I_2_-KI staining of pollen, pollen gains of soybean was observed under an OLYMPUS SE61 stereo microscope (Japan), and OLYMPUS CX31 microscope (Japan), respectively. Detection of pollen germination rate was performed according to Gai et al. [62] and observed under OLYMPUS CX31 microscope.

### 4.3. Sample Preparation and Extraction

The freeze-dried flower buds were crushed using a mixer mill (MM 400, Retsch Haan, Germany) with a zirconia bead for 1.5 min at 30 Hz. Then, 100mg powder was weighted and extracted overnight at 4 °C with 1.0 mL 70% aqueous methanol. Following centrifugation at 10,000× *g* for 10 min, the extracts were absorbed (CNWBOND Carbon-GCB SPE Cartridge, 250 mg, 3 mL; ANPEL, Shanghai, China) and filtrated (SCAA-104, 0.22 μm pore size; ANPEL, Shanghai, China) before LC-MS analysis.

### 4.4. UPLC-MS/MS Conditions

The sample extracts were analyzed using an ultra performance liquid chromatography coupled to tandem mass spectrometry system (UPLC: Shim-pack UFLC SHIMADZU CBM30A, Kyoto, Japan; MS/MS: Applied Biosystems 4500 Q TRAP, Foster City, CA, USA). The liquid conditions were as follows: UPLC column, Waters ACQUITY UPLC HSS T3 C18 (1.8 µm, 2.1 × 100 mm); solvent system, water (0.04% acetic acid): acetonitrile (0.04% acetic acid); gradient program, 95:5 (*v*/*v*) at 0 min, 5:95 (*v*/*v*) at 11.0 min, 5:95 (*v*/*v*) at 12.0 min, 95:5 (*v*/*v*) at 12.1 min, 95:5 (*v*/*v*) at 15.0 min; flow rate, 0.40 mL/min; temperature, 40 °C; injection volume: 5 μL. The effluent was alternatively connected to an ESI-triple quadrupole-linear ion trap (Q TRAP)-MS.

Linear ion trap (LIT) and triple quadrupole (QQQ) scans were acquired on a triple quadrupole-linear ion trap mass spectrometer (Q TRAP), API 4500 Q TRAP LC/MS/MS System, equipped with an ESI Turbo Ion-Spray interface, operating in a positive ion mode and controlled by Analyst 1.6.1 software (AB Sciex, Framingham, MA, USA). And the ESI source operation parameters were as follows: Ion source, turbo spray; source temperature 550 °C; ion spray voltage 5500 V; curtain gas was set at 25.0 psi; the collision gas was high. Instrument tuning and mass calibration were performed with 10 and 100 μmol/L polypropylene glycol solutions in QQQ and LIT modes, respectively. QQQ scans were acquired as multiple reaction monitoring (MRM) experiments. Declustering potential (DP) and collision energy (CE) for individual MRM transitions were done with further DP and CE optimization [63]. A specific set of MRM transitions were monitored for each period according to the metabolites eluted within this period.

### 4.5. Qualitative and Quantitative Determination of Metabolite

Mass spectrometry data were processed by Analyst 1.6.1 software. Based on the self-built database MWDB (Metware database) and the public database of metabolite information including METLIN (http://metlin.scripps.edu/index.php), MassBank (http://www.massbank.jp), and HMDB (http://www.hmdb.ca), the substance was qualitatively analyzed according to the secondary spectrum information. Isotope signals, repeated signals of K^+^, N^+^, NH^4+^ ions and fragment ions of other larger molecular weight substances were removed during the analysis.

Metabolite quantification was carried out via the MRM mode of QQQ mass spectrometry. In MRM mode, the quadrupole first screened the precursor ions (parent ions) of the target substance, and screened out the corresponding ions of other molecular weight substances to eliminate interference initially. The precursor ions were induced to ionize by collision chamber and then fragment ions break down to form many fragment ions, and then the fragment ions were filtered through the QQQ to select a required characteristic fragment ion, eliminating the interference of non-target ions. To screen out differential metabolites between NJCMS5A and NJCMS5B and to ensure the accuracy of the qualitative and quantitative analyses, the mass spectrum peak of each metabolite detected in different samples was corrected according to the retention time and peak shape of each metabolite [64].

### 4.6. Statistical Analysis

Metabolome data were analyzed using principal component analysis (PCA), repeated correlation assessment (RCA), orthogonal projections to latent structures-discriminant analysis (OPLS-DA), and heat map analysis. PCA results showed the trend of metabolites separation among NJCMS5A, NJCMS5B and mix, suggesting whether there were differences in metabolites between NJCMS5A and NJCMS5B [65,66]. Pearson’s correlation coefficient r was calculated using Perl (http://www.pm.org/) and used to evaluate the correlation between biological replicates. If r^2^ is closer to 1, the correlation between the two replicate samples is stronger. According to the OPLS-DA model, the metabolome data were analyzed and scoring map was drawn to further show the differences among the groups [67]. In the OPLS-DA model, the resulting R2X (the interpretability of the model on the categorical variable X), R2Y (the interpretability of the model on the categorical variable Y), and Q2 (the predictability of the model) were used to evaluate the validity of the model. Usually, a stable and reliable OPLS-DA model needs two factors, that is, the three indicators above are closer to 1 and Q2 > 0.9. The permutation test was performed multiple times (*n* = 200) to generate different R2’ and Q2’ values, which were used to further test model validity. If R2’ and Q2’ values are less than R2 and Q2 values of the OPLS-DA model, it shows that this model is meaningful. Two screening criteria for differential metabolites were established: The variable importance in the projection (VIP) ≥ 1 in the OPLS-DA model, and the |Log_2_FC (Fold Change)| ≥ 1. In order to observe the regularity of metabolite change conveniently, we normalized the metabolites with significant differences between NJCMS5A and NJCMS5B and drawn the cluster heat map using R software (R version 3.6.0, www.r-project.org/).

### 4.7. Kyoto Encyclopaedia of Genes and Genomes (KEGG) Annotation and Metabolic Pathway Analysis of Differential Metabolites

Differential metabolites were annotated using the KEGG database (http://www.kegg.jp/kegg/pathway.html) [68]. The classification and enrichment analysis of pathways with differential metabolites was performed to further identify key pathways related to metabolite differences.

### 4.8. Determination of ROS Scavenging Enzyme Activities and Flavonoid Content

SOD activity was measured at 550 nm on the vis spectrophotometer (722N, Jingke, Shanghai, China) using the plant total superoxide dismutase assay kit (Jiancheng, Nanjing, China). CAT activity was measured at 405 nm on the vis spectrophotometer using the plant catalase assay kit (Jiancheng, Nanjing, China). POD activity was measured at 420 nm on the vis spectrophotometer using the plant peroxidase assay kit (Jiancheng, Nanjing, China). Flavonoids content was measured at 502 nm on the micro plate reader (TECAN, infinite M200 PRO, Switzerland) using plant flavonoids assay kit (Jiancheng, Nanjing, China). All enzyme activity assay and flavonoid content analysis were performed for three biological replicates. Student’s *t*-test was used to determine significance of enzyme activity or flavonoids content differences between NJCMS5A and NJCMS5B.

## 5. Conclusions

This study was the first attempt to unravel secondary metabolites and antioxidant enzyme activity changes during flower bud development in soybean N8855-derived CMS plant. A UPLC-MS/MS-based metabolomics approach was performed to evaluate the metabolite differences between soybean CMS line and its maintainer. Of 612 metabolites, a total of 30 were up-regulated and 44 down-regulated in CMS line vs. its maintainer. We observed reduced flavonoids, phenolamides, and GSSG in soybean N8855-derived CMS line, which might have a negative effect on the scavenging of ROS. We also observed decreased activities of CAT and POD in CMS plant flower buds. Deficiencies in both enzymatic and non-enzymatic ROS scavenging systems may result in pollen abortion in soybean CMS-N8855.

## Figures and Tables

**Table 1:** Difference of flavonoids in flower buds of NJCMS5A and NJCMS5B.

Class	Index	Compounds	VIP	Fold Change	Type
Flavanone	Glm558	Naringenin *O*-malonylhexoside	1.61	2.51	up
	Glm656	Isoliquiritigenin	1.47	2.17	up
	Glm1180	Hesperetin 7-rutinoside	1.31	−22.16	down
	Glm1194	Hesperetin 7-*O*-neohesperidoside	1.52	−19.58	down
Flavone	Glm541	Apigenin *O*-malonylhexoside	1.52	2.28	up
	Glm559	Luteolin *O*-sinapoylhexoside	1.57	2.59	up
	Glm565	Tricetin *O*-malonylhexoside	1.46	2.19	up
	Glm578	Chrysin *O*-hexoside	1.92	3.76	up
	Glm590	Tricin *O*-rhamnoside	1.61	2.64	up
	Glm594	Chrysin 5-*O*-glucoside	2.00	4.28	up
	Glm1233	7,4′-Dihydroxyflavone	1.44	2.08	up
	Glm403	Selgin 5-*O*-hexoside	1.86	−3.42	down
	Glm694	Nobiletin	1.28	−12.40	down
	Glm710	Tangeretin	1.16	−17.82	down
	Glm1131	Tricin *O*-saccharic acid	1.77	−3.04	down
	Glm1169	Tricin 5-*O*-hexoside	1.38	−2.07	down
	Glm1182	Tricin di-*O*-hexoside	4.91	−5025.93	down
Flavone C-glycosides	Glm383	Isorhamnetin C-hexoside	3.95	247.04	up
	Glm536	Luteolin *O*-feruloylhexoside	1.66	2.71	up
	Glm278	8-C-hexosyl-hesperetin *O*-hexoside	5.17	−12,477.78	down
	Glm419	8-C-hexosyl-luteolin *O*-hexoside	1.42	−2.15	down
Flavonol	Glm587	Kaempferol 7-*O*-rhamnoside	1.39	2.27	up
	Glm1286	Syringetin	1.80	3.27	up
	Glm360	Syringetin 3-*O*-hexoside	1.63	−2.56	down
	Glm577	Quercetin-3,4′-*O*-di-β-glucopyranoside	3.36	−3033.67	down
	Glm640	Isorhamnetin	2.68	−3.06	down
	Glm1315	3,7-Di-*O*-methylquercetin	4.35	−794.44	down
Flavonolignan	Glm641	Tricin 4′-*O*-β-guaiacylglycerol	1.90	2.59	up
Isoflavone	Glm584	Daidzein	1.53	2.31	up
	Glm626	Genistein (4′,5,7-Trihydroxyisoflavone)	1.50	2.28	up
	Glm1212	6-Hydroxydaidzein	1.56	2.52	up
	Glm1249	Glycitein	1.71	2.80	up

**Table 2:** Difference of phenolamides in flower buds of NJCMS5A vs. NJCMS5B.

Index	Phenolamides	VIP	Fold Change	Type
Glm995	N-p-Coumaroyl hydroxyagmatine	1.35	2.13	up
Glm144	N-Caffeoylspermidine	2.45	−8.41	down
Glm207	N-p-Coumaroyl putrescine	3.66	−15.56	down
Glm224	N′-Feruloyl putrescine	2.67	−12.33	down
Glm228	N′-p-Coumaroyl putrescine	2.78	−9.23	down
Glm240	N′,N″,N‴-p-coumaroyl-cinnamoyl-caffeoyl spermidine	1.62	−2.58	down
Glm253	N-Feruloyl putrescine	2.76	−14.66	down
Glm255	N-Caffeoyl agmatine	3.30	−9.33	down
Glm270	N-p-Coumaroyl agmatine	1.40	−2.90	down
Glm281	N-Feruloyl agmatine	4.05	−50.00	down
Glm358	N′, N″-Feruloyl,caffeoylspermidine	2.07	−4.70	down
Glm377	N′, N″-di-p-coumaroylspermidine	2.90	−19.65	down
Glm576	N′,N″-p-coumaroyl-feruloyl putrescine	3.65	−108.53	down

## Supplementary Materials

The Supplementary Material for this article can be found online at https://www.mdpi.com/1422-0067/20/12/2869/s1.

## Abbreviations

AsA: Ascorbate; CAT: Catalase; CE: Collision energy; CMS: Cytoplasmic male sterility; DP: Declustering potential; GSH: glutathione; KEGG: Kyoto Encyclopaedia of Genes and Genomes; LIT: Linear ion trap; MS/MS: Tandem mass spectrometry; OPLS-DA: Orthogonal projections to latent structures-discriminant analysis; PCA: Principal component analysis; PCD: programmed cell death; POD: Peroxidase; QQQ: Triple quadrupole; RCA: Repeated correlation assessment; ROS: Reactive oxygen species; SEM: Scanning electron microscope; SOD: Superoxide dismutase; UPLC: Ultra Performance Liquid Chromatography; VIP: Variable importance in the projection.
