# Supplementary material for: Metabolomics Studies on Cytoplasmic Male Sterility during Flower Bud Development in Soybean
Source: Int J Mol Sci. 2019 Jun 12;20(12):2869. doi: 10.3390/ijms20122869 (PMC6627938; doi:10.3390/ijms20122869)
Supplement: Supplementary file 1 [file ijms-20-02869-s001.zip › Supplementary File/Supplementary Figure.pdf]

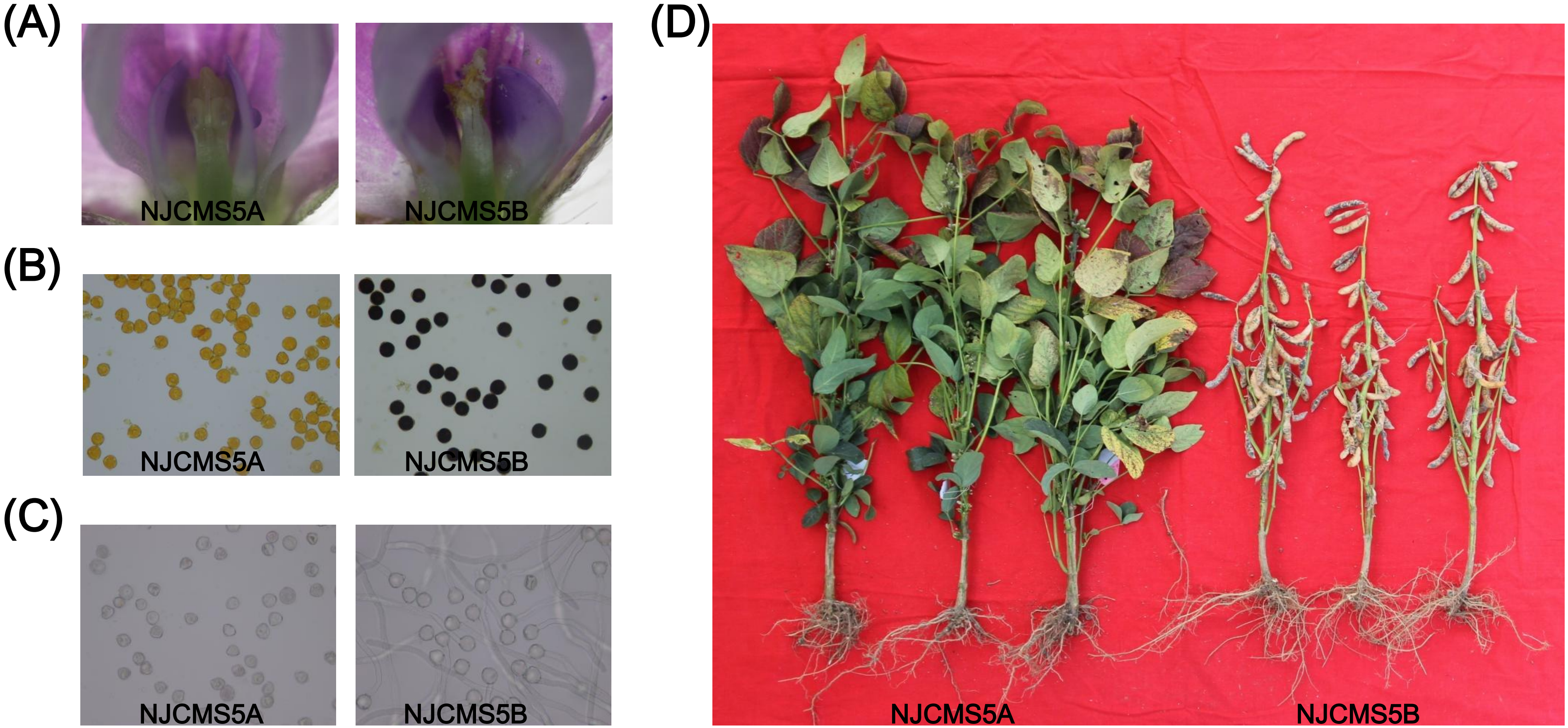

Figure S1 Morphological of anther, pollen and plant in NJCMS5A and NJCMS5B. (A) Anther dehiscence observed using 45× stereo microscope. **(B)** Pollen stained using 1% I_2-KI (100× microscope). **(C)** Pollen germination of NJCMS5A and NJCMS5B (100× microscope). **(D)** The plants of NJCMS5A and NJCMS5B during maturing in the field.
